# Comparison of [^18^F]DCFPyL and [^68^Ga]Ga-PSMA-HBED-CC for PSMA-PET Imaging in Patients with Relapsed Prostate Cancer

**DOI:** 10.1007/s11307-015-0866-0

**Published:** 2015-05-27

**Authors:** Markus Dietlein, Carsten Kobe, Georg Kuhnert, Simone Stockter, Thomas Fischer, Klaus Schomäcker, Matthias Schmidt, Felix Dietlein, Boris D. Zlatopolskiy, Philipp Krapf, Raphael Richarz, Stephan Neubauer, Alexander Drzezga, Bernd Neumaier

**Affiliations:** Department of Nuclear Medicine, University Hospital of Cologne, Kerpener Str. 62, 50937 Cologne, Germany; Institute of Radiochemistry and Experimental Molecular Imaging, University Hospital of Cologne, Cologne, Germany; Center of Integrated Oncology Cologne Bonn, University Hospital of Cologne, Cologne, Germany; Western German Prostata Center, “Klinik am Ring”, Cologne, Germany

**Keywords:** Prostate-specific membrane antigen (PSMA), F-18, Ga-68, Positron emission tomography (PET), Prostate cancer

## Abstract

**Purpose:**

Gallium-68 (Ga-68)-labeled tracers for imaging expression of the prostate-specific membrane antigen (PSMA) such as the [^68^Ga]Ga-PSMA-HBED-CC have already demonstrated high potential for the detection of recurrent prostate cancer. However, compared to Ga-68, a labeling with fluorine-18 (F-18) would offer advantages with respect to availability, production amount, and image resolution. [^18^F]DCFPyL is a promising F-18-labeled candidate for PSMA-positron emission tomography (PET) imaging that has been recently introduced. In the current study, we aimed to compare [^68^Ga]Ga-PSMA-HBED-CC and [^18^F]DCFPyL for clinical use in biochemically relapsed prostate cancer.

**Procedures:**

In 14 selected patients with PSA relapse of prostate cancer, [^18^F]DCFPyL PET/X-ray computed tomography (CT) was performed in addition to [^68^Ga]Ga-PSMA-HBED-CC PET/CT. A systematic comparison was carried out between results obtained with both tracers with regard to the number of detected PSMA-positive lesions, the standardized uptake value (SUV)_max_ and the lesion to background ratios.

**Results:**

All suspicious lesions identified by [^68^Ga]Ga-PSMA-HBED-CC were also detected with [^18^F]DCFPyL. In three patients, additional lesions were observed using [^18^F]DCFPyL PET/CT. The mean SUV_max_ in the concordant [^18^F]DCFPyL PSMA-positive lesions was significantly higher as compared to [^68^Ga]Ga-PSMA-HBED-CC (14.5 *vs.* 12.2, *p* = 0.028, *n* = 15). The mean tumor to background ratios (*n* = 15) were significantly higher for [^18^F]DCFPyL compared to [^68^Ga]Ga-PSMA-HBED-CC using kidney, spleen, or parotid as reference organs (*p* = 0.006, *p* = 0.002, *p* = 0.008), but no significant differences were found using the liver (*p* = 0.167) or the mediastinum (*p* = 0.363) as reference organs.

**Conclusion:**

[^18^F]DCFPyL PET/CT provided a high image quality and visualized small prostate lesions with excellent sensitivity. [^18^F]DCFPyL represents a highly promising alternative to [^68^Ga]Ga-PSMA-HBED-CC for PSMA-PET/CT imaging in relapsed prostate cancer.

## Introduction

The prostate-specific membrane antigen (PSMA) is overexpressed on the cell surface of prostate cancer (PC) cells [1]. Recent studies with gallium-68 (Ga-68)-labeled Glu-NH-CO-NH-Lys-(Ahx) ([^68^Ga]Ga-PSMA-HBED-CC) have shown the potential of this radioligand to detect relapses and metastases of PC with improved contrast when compared to [^18^F]fluoromethylcholine positron emission tomography (PET)/X-ray computed tomography (CT) [2–5]. Additionally, iodine-124, iodine-131, and lutetium-177-labeled PSMA ligands have been reported for dosimetric and therapeutic use [6, 7]. Several PSMA inhibitors were developed which compromise different pharmacophoric structures to interact with the binding pocket for *N*-acetyl-l-aspartyl-l-glutamate. PSMA-binding ligands are bound to the extracellular domain of PSMA. However, the transmembranous location of the binding domain and its enzyme activity enable the subsequent internalization of these ligands. The prostate-specific membrane antigen is also expressed in the tumor-associated neovasculature of gastric and colorectal cancer [8, 9]. Thus, these PSMA-selective ligands may be of interest for imaging other tumor types in the future.

In comparison to currently broadly applied Ga-68-labeled PSMA ligands, fluorine-18 (F-18)-labeled compounds would offer some important advantages. This includes not only an increase of the number of examinations owing to the higher production capacity but also an excellent image quality. The latter will be a result of optimized tracer doses leading to high imaging statistics and the decay properties of F-18 itself. F-18 exhibits a low positron emission energy of 0.6 MeV. Therefore, the distance to decelerate the positron in human tissue is much shorter in comparison to Ga-68 (β^+^-energy = 2.3 MeV) resulting in a much higher image resolution. Recently, Chen and colleagues [10] have published first data on [^18^F]DCFPyL (2-(3-{1-carboxy-5-[(6-[^18^F]fluoro-pyridine-3-carbonyl)-amino]-pentyl}-ureido)-pentanedioic acid), a new PSMA-selective ligand with a high binding affinity for PSMA. It was suggested that this compound may represent a highly promising candidate for PET imaging of PSMA-overexpressing tissues [10].

In this work, a comparison between [^68^Ga]Ga-PSMA-HBED-CC and [^18^F]DCFPyL PET/CT in patients with a PSA-relapse of prostate cancer was carried out.

We assumed that [^18^F]DCFPyL would bear the potential of high diagnostic accuracy due to the following:

- Availability of higher tracer activity amounts in combination with the longer half-life of F-18, enabling imaging at later time points resulting in higher clearance and lower nonspecific binding
- Higher image quality due to lower positron emission energy of F-18

We aimed to confirm and potentially extend the findings obtained with our diagnostic standard, [^68^Ga]Ga-PSMA-HBED-CC PET/CT, by adding a second scan with [^18^F]DCFPyL, representing a different PSMA tracer. By choosing this approach, we tried to collect accumulating evidence in the diagnostic assessment of selected patients and to compare the properties of [^18^F]DCFPyL with [^68^Ga]Ga-PSMA-HBED-CC at the same time.

## Materials and Methods

### Patient Characteristics

In this study, 14 selected patients were included who underwent both [^68^Ga]Ga-PSMA-HBED-CC PET/CT and [^18^F]DCFPyL PET/CT with the aim to determine if one of the two tracers exhibits better detection of recurrent prostate cancer and/or metastases. The patients had biochemical relapse of prostate cancer after initial curative treatment, either radical prostatectomy or radiation-based therapy. All patients showed rising PSA level and suspected progressive disease following prior treatment of prostate cancer. In 11 patients, the PSA level had increased to more than 1 ng/ml. All patients underwent the examination as part of their clinical workup. Patients were selected for the dual-scan procedure assuming that a thorough diagnostic assessment would have a significant influence on their individual subsequent therapeutic measures, *i.e.*, local *versus* systemic therapy, indication for or exclusion from surgical treatment or external beam radiation therapy. This included, *e.g.*, suspected false-negative or equivocal PET/CT results, detection of a solitary metastasis, or oligometastatic status potentially accessible for local therapy.

First, [^68^Ga]Ga-PSMA-HBED-CC PET/CT was performed, representing the standard procedure at our center. Subsequently, in 14 selected patients, additional [^18^F]DCFPyL PET/CT was carried out within a period of 3 weeks following the first scan. All patients underwent the examinations as part of the clinical workup in order to accumulate diagnostic evidence and potentially optimize their individual treatment. This study does not represent a systematic clinical trial, comparing the clinical value of two diagnostic instruments in a blinded fashion. All patients signed informed consent regarding the scientific evaluation of their data.

### Preparation of [^68^Ga]Ga-PSMA-HBED-CC

[^68^Ga]Ga-PSMA-HBED-CC was produced according to the methods reported by Eder *et al.* [11] and Schäfer *et al.* [12]. The whole labeling procedure was carried out under good manufacturing practice (GMP) conditions. For labeling, a ^68^Ge/^68^Ga-generator (iThemba), distributed by IDB Holland BV (Baarle-Nassau, the Netherlands), was used. After fractionated elution with 5 ml 0.6 M HCl, 500 μl Ga-68 generator eluate were neutralized with 250 μl 2 M sodium acetate solution (in Ultrapur water, sterilized in 1 ml aliquots). Then, 500 μl (5 μg in water, Ultrapur, Merck, Darmstadt, Germany) of PSMA-HBED-CC (ABX, Radeberg, Germany; CA index name: 4,6,12,19-tetraazadocosane-1,3,7-tricarboxylic acid, 22—[3-[[[2-[[[5-(2-carboxyethyl)-2-hydroxyphenyl] -methyl] (carboxymethyl) amino] ethyl] (carboxymethyl) amino] methyl]-4-hydroxyphenyl]—5,13,20-trioxo-, (3S,7S)-, supplied as trifluoroacetate salt) were added and mixed vigorously. The reaction mixture was heated at 80 °C for 10 min. After 5 min cooling at room temperature (RT), the solution was diluted with 8.75 ml sterile isotonic saline. Two aliquots for quality controls (radiochemical purity and endotoxin testing) were withdrawn. Radiochemical purity was determined by reversed phase HPLC (WellChrom system, Knauer, Berlin, Germany) with a Knauer Hypersil ODS (4 mm) column. Endotoxin testing (LAL) was carried out on Endosafe MCS™ from Charles River (Ecully, France) using their cartridges with a sensitivity of 5.0–0.05 EU/ml. Samples were checked for sterility by the Institute for Medical Microbiology, Immunology and Hygiene, University Hospital Cologne. Labeling provided [^68^Ga]Ga-PSMA-HBED-CC in high radiochemical purity (>98 %). The specific activity of [^68^Ga]Ga-PSMA-HBED-CC was 62 GBq/μmol.

In addition, the affinity-related IC_50_ values of the reaction mixtures of both labeling conditions were determined on the PSMA expressing cell line LNCaP. Indeed, both the RT-labeled fraction and the 95 °C-labeled fraction bound PSMA with identical affinities (IC_50_ values 27.4 ± 1.3 and 24.8 ± 1.2 nm, respectively) [13].

### Preparation of [^18^F]DCFPyL

[^18^F]Fluoride was produced *via* the ^18^O (p,n)^18^F reaction by bombardment of enriched [^18^O] water with 16.5 MeV protons using a MC16 cyclotron (Scanditronix, Uppsala, Sweden) at the Max Planck Institute for Metabolism Research.

The synthesis of [^18^F]DCFPyL was performed under GMP conditions as previously reported by Chen *et al.* [10]. The radiolabeled product was analyzed using the following conditions: column: Chromolith SpeedROD®, 50 × 4.6 mm (Merck Millipore, Darmstadt, Germany); eluent: 5 % EtOH in 0.38 % H_3_PO_4_ (pH 2); flow rate: 3.0 ml/min; *t*_*r*_ = 2.2 min. The final product was formulated in a PBS solution (pH 4–6). The formulated solution of [^18^F]DCFPyL was tested for sterility and endotoxin content. Production under GMP conditions provided [^18^F]DCFPyL in reasonable radiochemical yields of 8–12 % and in high radiochemical purity (>98 %). The specific activity of [^18^F]DCFPyL amounted to 72 GBq/μmol.

The PSMA enzyme inhibition potency of compound 3 was determined with a modified Amplex Red glutamic acid assay after incubation with the cell lysates of LNCaP cell extracts in the presence of NAAG for 2 h at 37 °C. The enzyme inhibitory constant (Ki) for compound 3 was 1.1 ± 0.1 nmol/l, comparable with that of ZJ-43, which was 1.4 ± 0.2 nmol/l under same measurement conditions. ZJ-43 is a urea-based potent inhibitor of NAAG and is used as an internal reference in the assay.

### Imaging

The mean dosage of 128.3 MBq ± 35.9 MBq [^68^Ga]Ga-PSMA-HBED-CC solution was injected as an intravenous bolus. Variation of the injected activity was caused by the variable elution efficiencies obtained during the lifetime of the ^68^Ge/^68^Ga radionuclide generator. The preparations contained 2.65 nmol PSMA ligand.

The mean dosage of 318.4 MBq ± 59.0 MBq [^18^F]DCFPyL complex solution was injected as an intravenous bolus. The variability of the injected dose of [^18^F]DCFPyL is explained by the available activity after the production of [^18^F]DCFPyL and by adaption to body weight. The preparations contained between 2.7 and 5.1 nmol (mean 4.0 nmol) PSMA ligand depending on the dosage of [^18^F]DCFPyL.

Patients fasted for at least 4 h prior to injection of [^68^Ga]Ga-PSMA-HBED-CC or [^18^F]DCFPyL. There were no adverse effects observed in any of the patients after injection of both tracers.

For both tracers, whole-body PET and a non-contrast-enhanced (low-dose) CT scan were performed on a Biograph 16 PET/CT scanner (Siemens, Erlangen, Germany) in eight patients and on a Biograph mCT 128 scanner (Siemens, Erlangen, Germany) in six patients 1 h post injection of the [^68^Ga]Ga-PSMA-HBED-CC and 2 h post injection of the [^18^F]DCFPyL, respectively. The time window between injection and start of data acquisition was chosen analogously to published data for [^68^Ga]Ga-PSMA-HBED-CC [2–5] and for [^18^F]DCFPyL [14]. Attenuation correction was performed using the low-dose non-enhanced CT data.

To ensure comparability between different PET/CT scanners, reconstruction was performed *via* an OSEM algorithm (4 iterations and 14 subsets), followed by an intrinsic 5-mm Gaussian filter in all directions for the Biograph 16 True Point (Siemens Medical Solutions), containing full-ring dedicated PET and 16-slice CT instrumentation. When using the Biograph mCT Flow-Edge 128 PET/CT system (Siemens Medical Solutions) with a 128-slice spiral CT, iterative reconstruction was performed using 4 iterations and 12 subsets by an intrinsic 5-mm Gaussian filter.

### Image Analysis

Image analysis was performed using an appropriate workstation and software (Syngo TrueD, Siemens, Erlangen, Germany). Clinical PET/CT reading was performed by three experienced specialists from the department of nuclear medicine and from the department of radiology in consensus in a side-by-side analysis of the results obtained with both tracers. Lesions were visually interpreted as suspicious for local relapse, lymph node metastasis, bone metastasis, or visceral metastasis.

The standardized uptake value (SUV)_max_ was measured in up to three hottest lesions (as identified in the [^68^Ga]Ga-PSMA-HBED-CC scan) and their counterpart in the [^18^F]DCFPyL scan. Background SUV_mean_ values were measured in a volume of interest (VOI) with 2 cm diameter in the liver, spleen, kidney, mediastinum, and parotid in all patients. For the calculation of mean values and to compare the SUV_max_ values of the lesions and their ratios, SPSS 22 was used.

## Results

Patient characteristics are shown in Table 1.

**Table 1:** Patient characteristics and pathological tracer uptake in [^68^Ga]Ga-PSMA-HBED-CC PET/CT and [^18^F]DCFPyL PET/CT. The preparations contained between 2.7 and 5.1 nmol (mean 4.0 nmol) of the F-18-labeled PSMA ligand depending on the dosage of [^18^F]DCFPyL, and 2.65 nmol of the Ga-68-labeled PSMA ligand

Patient no.	Age (years)	PSA (ng/ml)	^68^Ga dosage ^18^F dosage (MBq)	Local PSMA +	Nodal PSMA +	Distant PSMA +	Therapeutic consequence	Verification
1 [^68^Ga]PSMA [^18^F]PSMA	53	4.1	177 350	1 1	1 1	0 0	Surgery	LN histologically confirmed. LR not confirmed by core biopsy
2 [^68^Ga]PSMA [^18^F]PSMA	74	4.7	138 364	0 0	0 0	2 3	Systemic and RT of bone metastases	CT
3 [^68^Ga]PSMA [^18^F]PSMA	86	2.1	139 347	1 1	0 0	0 0	Core biopsy of prostate	n.a.
4 [^68^Ga]PSMA [^18^F]PSMA	68	n.a.	104 411	1 1	0 0	0 0	Core biopsy of prostate	LR not confirmed by core biopsy
5 [^68^Ga]PSMA [^18^F]PSMA	82	50	139 382	0 0	>10 >10	0 0	Systemic	CT
6 [^68^Ga]PSMA [^18^F]PSMA	66	1.24	163 319	0 0	0 0	0 0	Wait and see	No target for biopsy
7 [^68^Ga]PSMA [^18^F]PSMA	68	1.3	139 349	0 0	1 2	0 0	Surgery	1 LN histologically confirmed
8 [^68^Ga]PSMA [^18^F]PSMA	80	1.2	127 216	0 0	0 0	0 0	Wait and see	No target for biopsy
9 [^68^Ga]PSMA [^18^F]PSMA	72	0.17	74 310	0 0	0 0	0 0	Wait and see	No target for biopsy
10 [^68^Ga]PSMA [^18^F]PSMA	68	1.2	110 240	1 1	0 0	0 0	Core biopsy of prostate	n.a.
11 [^68^Ga]PSMA [^18^F]PSMA	60	2.04	95 280	0 0	1 1	0 0	RT or surgery	n.a.
12 [^68^Ga]PSMA [^18^F]PSMA	74	3.87	64 360	0 0	5 10	0 2	Systemic	CT (bone)
13 [^68^Ga]PSMA [^18^F]PSMA	51	0.4	140 232	0 0	0 0	0 0	Wait and see	No target for biopsy
14 [^68^Ga]PSMA [^18^F]PSMA	54	10	187 297	0 0	2 2	0 0	RT or surgery	n.a.

*LN* lymph node, *LR* local relapse, *n.a.* not available, *RT* radiotherapy, *[* ^*68*^ *Ga]PSMA* [^68^Ga]Ga-PSMA-HBED-CC PET/CT, *[* ^*18*^ *F]PSMA* [^18^F]DCFPyL PET/CT

### Presence of PSMA-Positive Lesions

In 4 of the 14 patients (29 %), no PSMA-positive lesions were detected with either [^68^Ga]Ga-PSMA-HBED-CC or with [^18^F]DCFPyL. In 10 of 14 patients (71 %), at least one lesion suspicious for prostate cancer tissue was detected in [^68^Ga]Ga-PSMA-HBED-CC PET/CT imaging and in [^18^F]DCFPyL PET/CT imaging. In 3 patients, at least one additional PSMA-positive lesion was detected with [^18^F]DCFPyL PET/CT as compared to the [^68^Ga]Ga-PSMA scan. Thus, all patients were classified accordingly with both tracers regarding the general presence of a PSMA-positive suspicious finding (yes/no). Eleven patients (79 %) would also have been classified consistently with both tracers regarding number and location of their lesions, and in 3 (21 %) of patients, additional lesions were detected with [^18^F]DCFPyL.

### Type and Location of Lesions

With respect to the type/location of suspicious lesions, [^68^Ga]Ga-PSMA-HBED-CC PET/CT had detected 2 bone metastases, and [^18^F]DCFPyL PET/CT had detected 5 putative bone metastases. [^68^Ga]Ga-PSMA-HBED-CC PET/CT had detected 20 lymph node metastases, and [^18^F]DCFPyL PET/CT had detected 26 putative lymph node metastases. [^68^Ga]Ga-PSMA-HBED-CC PET/CT and [^18^F]DCFPyL PET/CT were suspicious for local relapse in 4 cases each.

The additional suspicious lesions detected with [^18^F]DCFPyL were found in the lumbar vertebra L2 in a patients with bone metastases in the left humerus and the right femur (patient 2), in a pelvic lymph node beyond the vena cava inferior in a patient with a concordant left iliac PSMA-positive lymph node metastasis (patient 7), and in mediastinal, left cervical, left supraclavicular, left axillary lymph nodes as well as in the thoracal vertebra Th3 and the sternal *processus xiphoideus* in a patient with concordant PSMA-positive retroperitoneal lymph node metastases (patient 12).

### Verification of Lesions

**Fig. 1:**
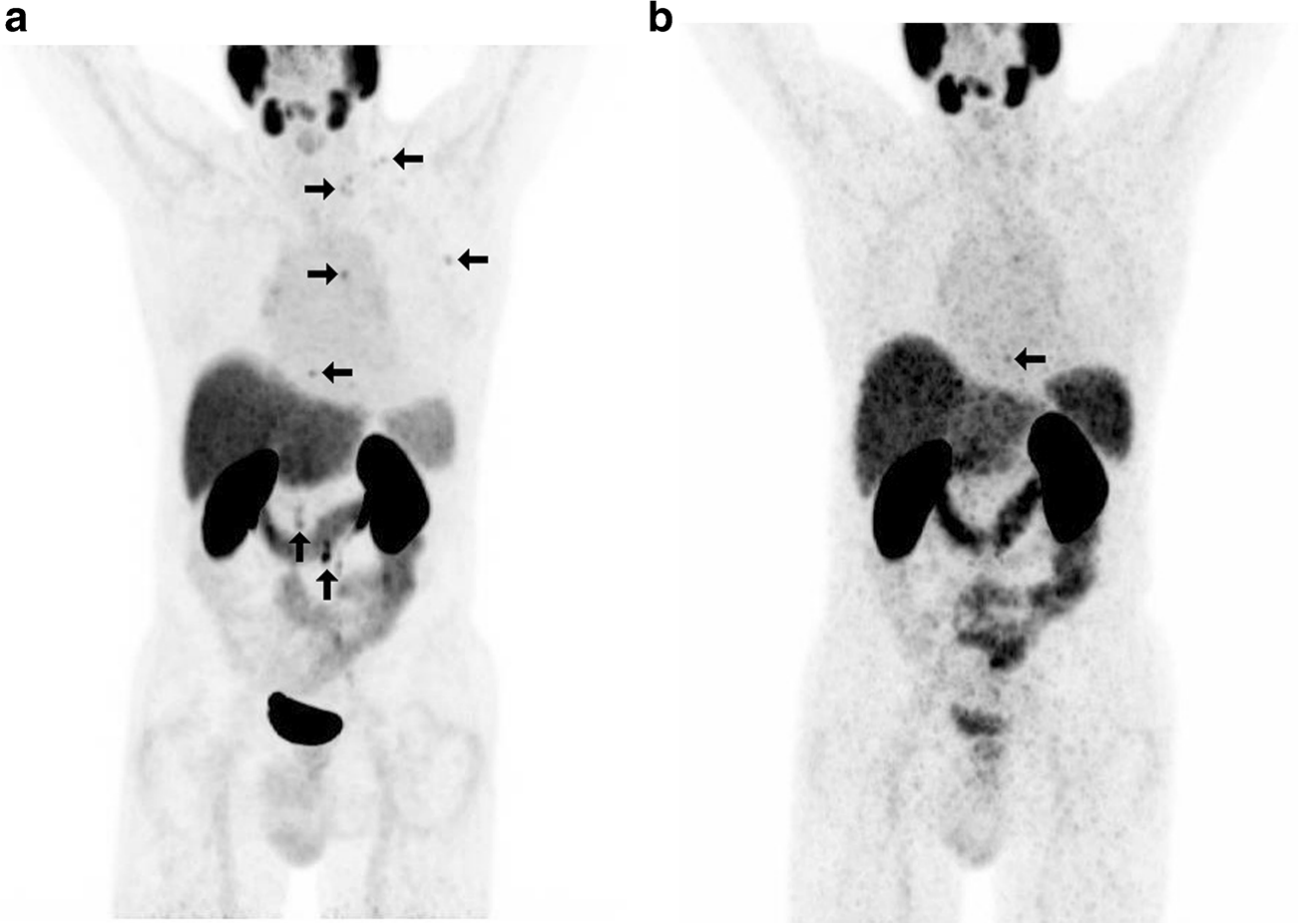
Patient no. 12 with a rising PSA level of 3.87 ng/ml. In the past, the patient had retroperitoneal lymph node metastases, which were irradiated. On a Biograph mCT 128 scanner, comparison between **a** MIP (maximum intensity projection) with [^18^F]DCFPyL and **b** MIP with [^68^Ga]Ga-PSMA-HBED-CC. [^18^F]DCFPyL PET/CT clearly demonstrates several additional supradiaphragmatic PSMA-positive lesions. [^68^Ga]Ga-PSMA-HBED-CC PET/CT showed the supradiaphragmal lesion in the sternum.

**Fig. 2:**
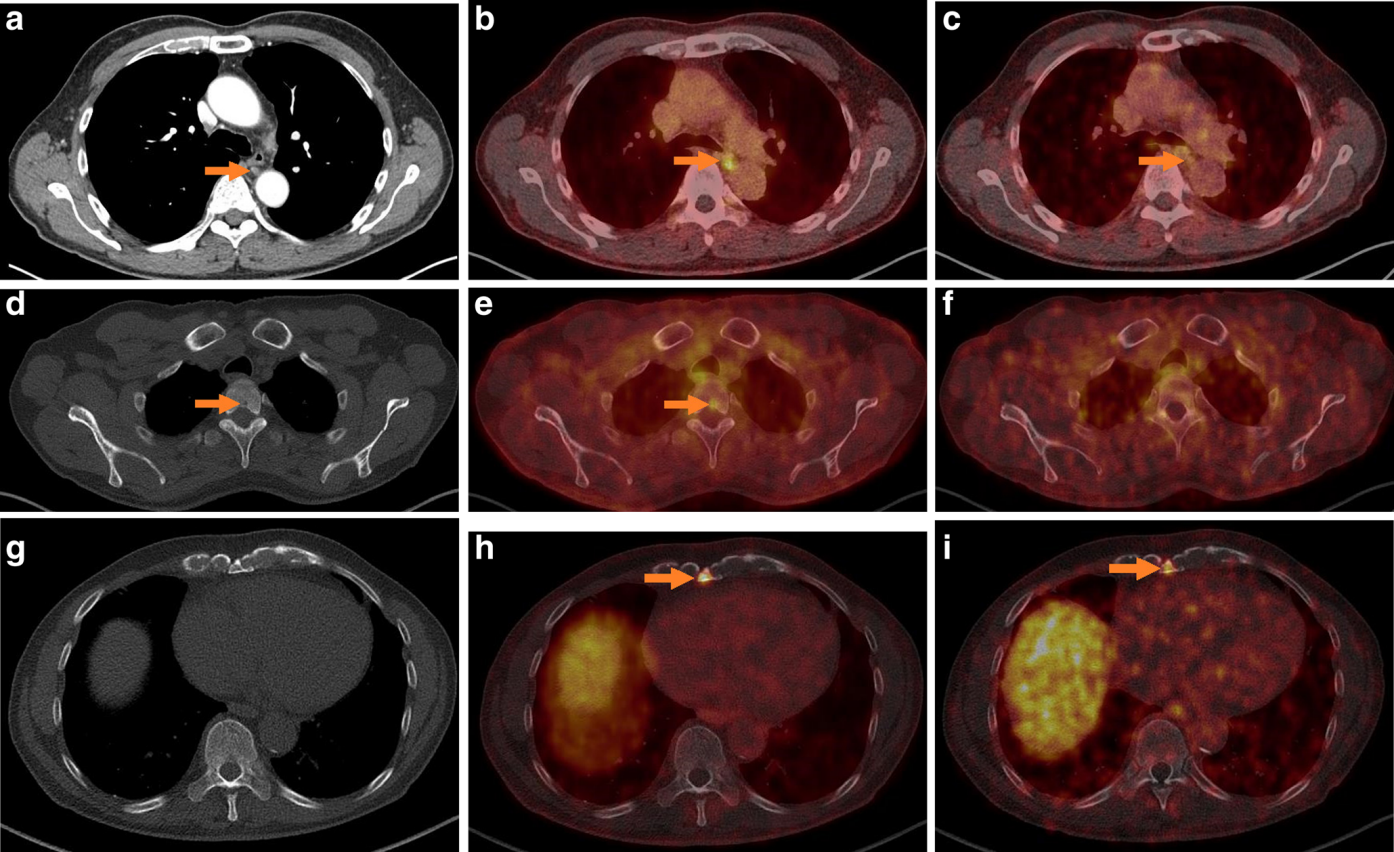
Patient no. 12, Biograph mCT 128. [^18^F]DCFPyL PET/CT (**b**) was suspicious of an additional lymph node metastasis in the dorsal mediastinum, visible as a normal-sized lymph node in **a** low-dose CT scan without tracer accumulation on **c** [^68^Ga]Ga-PSMA-HBED-CC PET/CT. **d** Low-dose CT in bone window and **e** [^18^F]DCFPyL PET/CT were suspicious of a bone metastasis in vertebra Th3, barely detectable in **f** [^68^Ga]Ga-PSMA-HBED-CC PET/CT. **h** [^18^F]DCFPyL PET/CT was clearly suspicious of bone metastasis in the *processus xiphoideus sterni*, hard to detect in **g** low-dose CT or **i** [^68^Ga]Ga-PSMA-HBED-CC PET/CT.

**Fig. 3:**
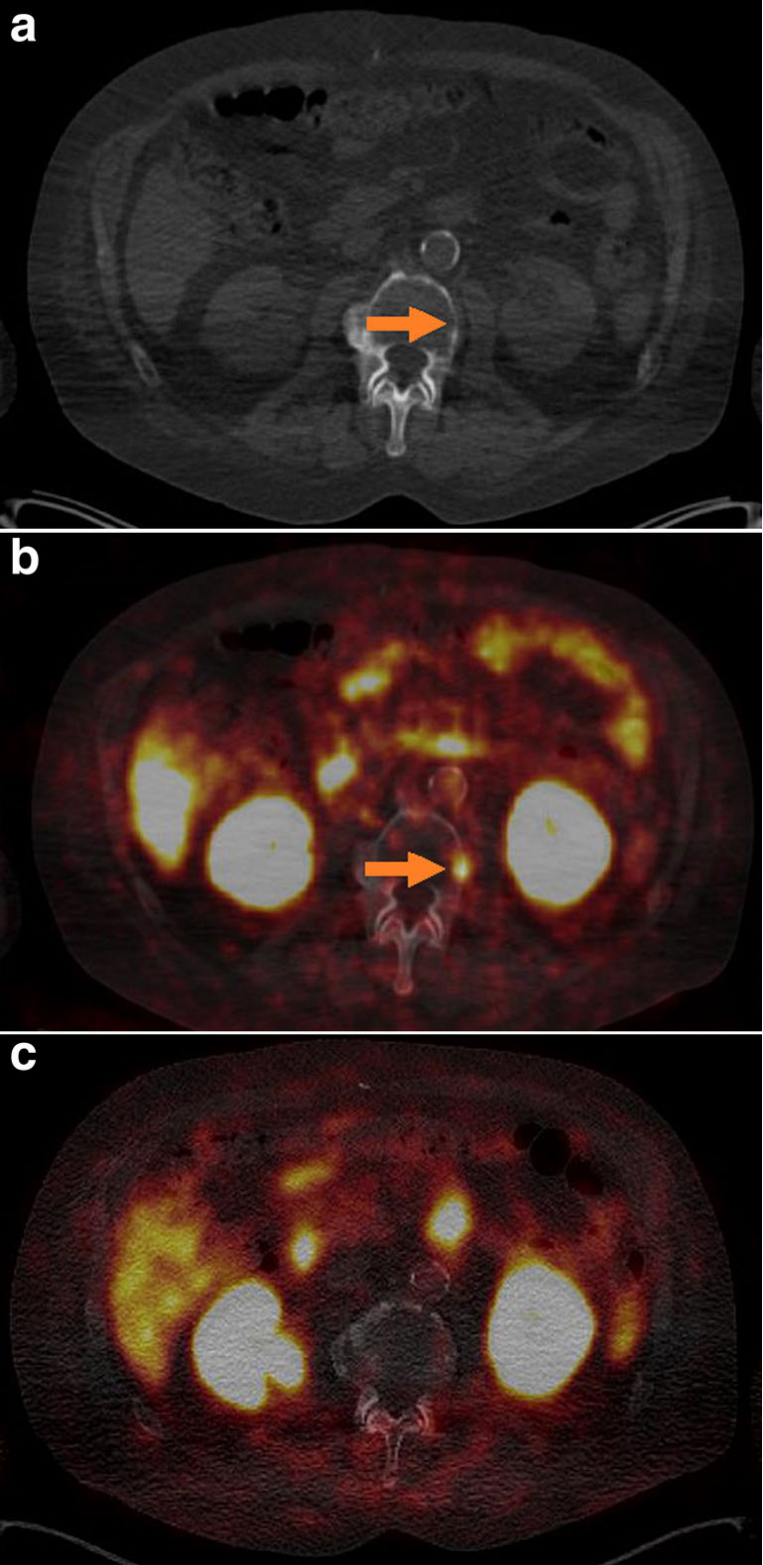
Patient no. 2 with a rising PSA level of 4.7 ng/ml. **a** Low-dose CT in bone window and **b** [^18^F]DCFPyL PET/CT were suspicious of bone metastasis in vertebra L2, hard to discern in the **c** [^68^Ga]Ga-PSMA-HBED-CC PET/CT. Imaging was performed on a Biograph 16 PET/CT.

**Fig. 4:**
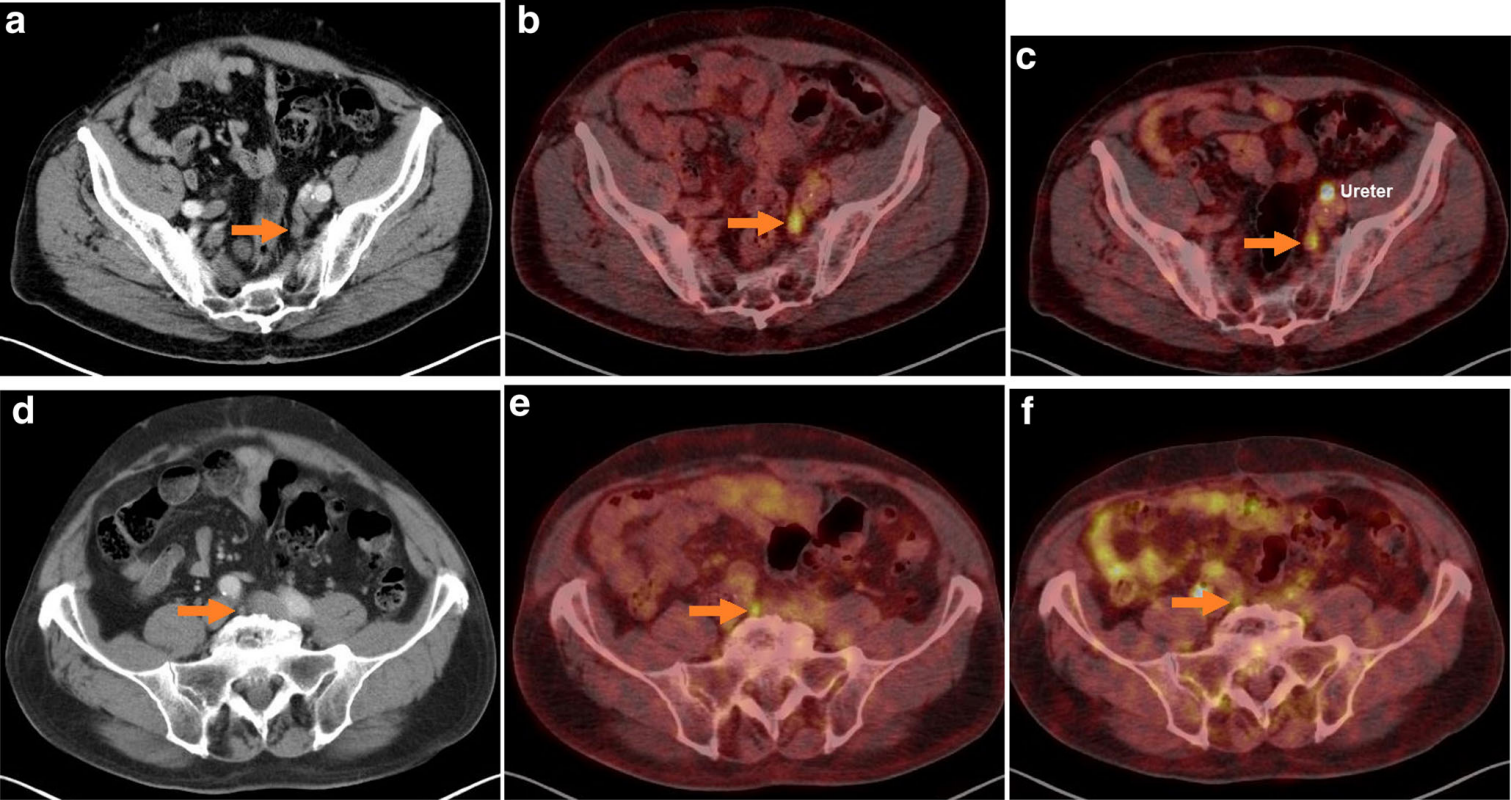
Patient no. 7 with a rising PSA level to 1.3 ng/ml. In the past, the patient had left iliac lymph node dissection with histologically confirmed lymph node metastases. Both tracers have shown a left iliac PSMA-avid lymph node, which was confirmed as metastasis (**a**–**c**). Additionally, **e** the [^18^F]DCFPyL PET/CT was suspicious of a second PSMA-positive lymph node below the bifurcation of the v. cava inferior, visible as a normal-sized lymph node on **d** CT, hard to detected on **f** [^68^Ga]Ga-PSMA-HBED-CC PET/CT. **a** Low-dose CT scan, **b** [^18^F]DCFPyL PET/CT, and **c** [^68^Ga]Ga-PSMA-HBED-CC PET/CT at the level of the *iliacae communes.*_**d** Low-dose CT scan, **e** [^18^F]DCFPyL PET/CT, and **f** [^68^Ga]Ga-PSMA-HBED-CC PET/CT at the level of the bifurcation*.* Imaging was performed on a Biograph 16 PET/CT.

The bone metastases in the lumbar vertebra L2 (patient 2) and in the thoracal vertebral Th3 (patient 12) were confirmed by CT retrospectively. In patient 7, a concordant left iliac PSMA-positive lymph node was detected with both tracers and an additional F-18 PSMA-positive pelvic lymph was reported. This patient underwent surgical lymph node resection, and the left iliac metastasis was confirmed histologically. The additional pelvic metastasis was not detected by salvage lymph node dissection, but PSA remained increased postoperatively, indicating that this lesion may have represented the remaining PSA-producing tissue. In 1 patient, the solitary PSMA spot in the irradiated prostate, seen in the [^68^Ga]Ga-PSMA-HBED-CC PET/CT and in the [^18^F]DCFPyL PET/CT, was not confirmed by core biopsy (patient 4). In 1 patient, the concordant solitary PSMA-avid lymph node metastasis was confirmed histologically by systematic lymphadenectomy, while core biopsy was negative for local relapse despite a focal PSMA concentration within the irradiated prostate (patient 1). Examples of the PSMA-positive lesions are shown in Figs. 1, 2, 3, and 4.

### Quantitative Comparison

The mean SUV_max_ in the concordant PSMA-positive lesions was 14.5 for [^18^F]DCFPyL and 12.2 for [^68^Ga]Ga-PSMA-HBED-CC (*p* = 0.028, *n* = 15 metastases). The mean background SUV_mean_ values for [^18^F]DCFPyL and [^68^Ga]Ga-PSMA-HBED-CC in all 14 patients examined were 6.2 and 5.1 in the liver (*p* = 0.049), 4.9 and 7.2 in the spleen (*p* = 0.005), 19.6 and 31.7 in the kidney (*p* = 0.001), 1.3 and 1.2 in the mediastinum (*p* = 0.056, n.s.), and 10.9 and 12.9 in the parotid (*n* = 0.121, n.s.), respectively.

**Fig. 5:**
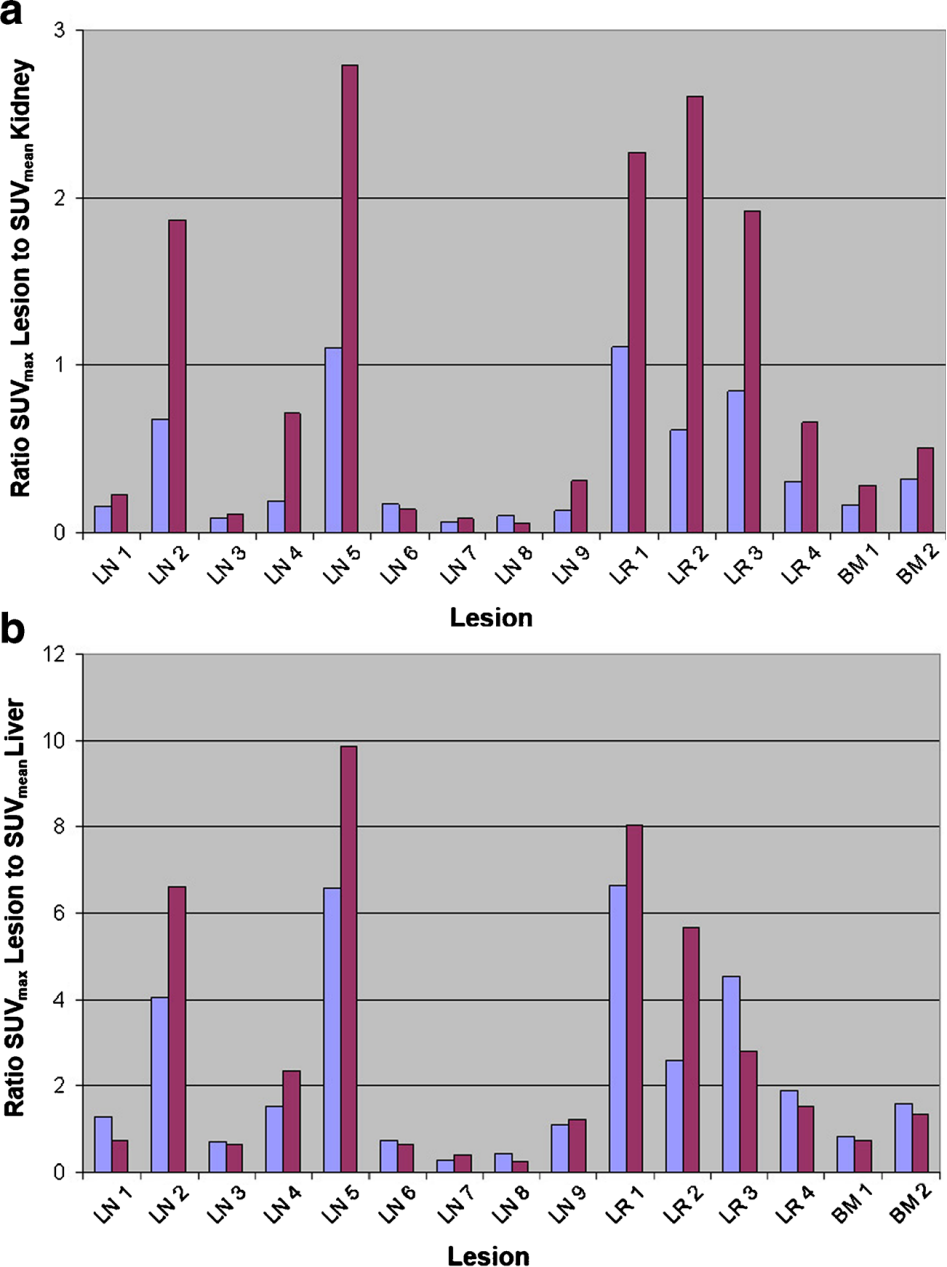
**a** Ratio of SUV_max_ in lesions to SUV_mean_ in the kidney and **b** ratio of SUV_max_ in lesions to SUV_mean_ in the liver. The *blue columns* represent [^68^Ga]Ga-PSMA-HBED-CC PET/CT, and the *red columns* represent [^18^F]DCFPyL PET/CT. [^18^F]DCFPyL showed a higher tumor to background ratio than [^68^Ga]Ga-PSMA-HBED-CC when the kidney was used as a reference organ (*p* = 0.006, *n* = 15). The tumor to background ratio did not differ significantly between [^18^F]DCFPyL and [^68^Ga]Ga-PSMA-HBED-CC when the liver was used as a reference organ (*p* = 0.167, *n* = 15). *LN* lymph node metastasis, *LR* local relapse, *BM* bone metastasis.

To compare the tumor/background contrast between the two tracers, we calculated SUV values in suspicious lesions/background in seven patients with maximum three lesions, detectable with both PET procedures. This resulted in a comparison of altogether 15 tumor/background ratios. Most of the suspected lymph node/bone metastases showed a higher SUV ratio for [^18^F]DCFPyL using the kidney (Fig. 5a) or the spleen as a reference region, but no significant difference was found when using for the liver (Fig. 5b) or the mediastinum as reference organ. In detail, the mean tumor to background ratios between the SUV_max_ in the PSMA-avid lesions with [^18^F]DCFPyL and [^68^Ga]Ga-PSMA-HBED-CC were 2.9 and 2.3 as compared with the SUV_mean_ in the liver (*p* = 0.167, n.s.), 4.3 and 2.1 as compared with the SUV_mean_ in the spleen (*p* = 0.002), 1.0 and 0.4 as compared with the SUV_mean_ in the kidney (*p* = 0.006), 10.7 and 9.7 as compared with the SUV_mean_ in the mediastinum (*p* = 0.363, n.s.), and 1.4 and 0.9 as compared with the SUV_mean_ in the parotid (*p* = 0.008), respectively.

### Clinical Consequences

In the majority of cases, [^68^Ga]Ga-PSMA-HBED-CC and [^18^F]DCFPyL PET/CT resulted in accumulating evidence, supporting further treatment decisions consistently. Both imaging series accordingly supported decisions towards core biopsy of the irradiated prostate in three patients, to local therapy options (lymph node dissection or radiotherapy) in four patients with one to two PSMA-positive lymph nodes and to systematic therapy in three patients. In four patients, neither imaging test detected a lesion explaining the rise in PSA levels.

[^18^F]DCFPyL PET/CT resulted in the detection of additional vertebral bone metastases in two patients (patient 2 and 12) in an oligometastatic state (patient 2 showed bone metastases also in [^68^Ga]Ga-PSMA-HBED-CC PET/CT but to a lesser extent). This finding did not have immediate therapeutical consequences because both patients were asymptomatic at the time of their PET/CT examination. However, more accurate knowledge on the extent of bone metastases bears the potential to perform better targeted radiotherapy in the future (*i.e.*, if the patients become symptomatic from their bone metastases). The detection of many PSMA-positive supradiaphragmatic lymph nodes with [^18^F]DCFPyL in addition to retroperitoneal lymph node metastases also detected with [^68^Ga]Ga-PSMA-HBED-CC supported the decision to omit local radiotherapy of the retroperitoneal lymphatic pathways (patient 12). In one patient, a second PSMA-avid lymph node in the upper pelvis was detected with [^18^F]DCFPyL in addition to another pelvic lymph node (detected with both tracers). In this patient, systematic bilateral pelvic lymph node dissection was performed and not influenced by the [^18^F]DCFPyL findings. Unfortunately, only the larger metastasis (positive with both tracers) was detected intraoperatively and the patient remained PSA positive after surgery (patient 7).

## Discussion

We analyzed 14 patients who underwent both [^68^Ga]Ga-PSMA-HBED-CC PET/CT and [^18^F]DCFPyL PET/CT. In all cases, a rising PSA level indicated disease recurrence following prior treatment of prostate cancer.

The following findings emerge from this analysis:

- All suspicious lesions identified with [^68^Ga]Ga-PSMA-HBED-CC were detected with [^18^F]DCFPyL consistently.
- [^18^F]DCFPyL demonstrated a significantly higher SUV_max_ as compared to [^68^Ga]Ga-PSMA-HBED-CC in consistent lesions and a better tumor to background ratio when the kidney, spleen, or parotid was used as a reference organ. The tumor to background ratio did not differ significantly between [^18^F]DCFPyL and [^68^Ga]Ga-PSMA-HBED-CC when the liver or the mediastinum was used as a reference organ. Although based on a small number of subjects, these findings support the notion that [^18^F]DCFPyL provides at least comparable tumor/background contrast as observed with [^68^Ga]Ga-PSMA-HBED-CC.
- Using [^18^F]DCFPyL, additional suspicious lesions plausible for further metastases were detected in 3/14 patients. This indicates a high sensitivity of this tracer.

One explanation for the higher detection rate using [^18^F]DCFPyL can be found in the higher injected dose, which allowed later acquisition times, potentially leading to better signal to noise ratios due to reduction of nonspecific signal. Lower background activity has been observed for the [^18^F]DCFPyL in some organs such as the kidney. Independently from injected dose, faster clearance of the tracer from non-target tissue and higher affinity, may potentially have contributed to the detection of additional skeletal metastases observed for [^18^F]DCFPyL as compared to [^68^Ga]Ga-PSMA-HBED-CC.

It is important to notice that the higher detection rate for PSMA-avid lymph nodes by [^18^F]DCFPyL was not attributed to retroperitoneal PSMA foci, where the differentiation against celiac ganglia (*i.e.*, false-positive findings) might be a question [15].

Radiolabeling with Ga-68 is an excellent alternative for imaging centers with expertise in handling the commercially available ^68^Ge/^68^Ga radionuclide generator but without own (cost-intense) cyclotron. However, a single preparation (0.3–0.6 GBq Ga-68) may only allow scanning up to two to four patients and the output depends on the half-life of the generator. As now established at our center, a single radiolabeling procedure of [^18^F]DCFPyL resulted in a batch containing 6 to 7 GBq of [^18^F]DCFPyL. This rendered it possible to examine six patients after a single preparation with an optimal dose, which represents an advantage for centers with an own cyclotron and a production of F-18 in the daily management. Furthermore, due to the longer half-life of F-18 (110 *vs.* 68 min for Ga-68), it may also allow transportation to remote sites from commercial vendors.

A limitation of the current study is the lack of a consistent histological validation of [^18^F]DCFPyL findings. In principle, it cannot be excluded that additional lesions detected with [^18^F]DCFPyL may represent false-positive findings. However, particularly small PSMA-positive findings cannot be easily confirmed histologically and it is impossible to assess all lesions in patients with multiple metastases. Generally, good correspondence between [^18^F]DCFPyL and [^68^Ga]Ga-PSMA-HBED-CC findings was observed, several lesions were confirmed histopathologically, and the additional bone lesions detected with [^18^F]DCFPyL were verified retrospectively by CT findings suspicious for bone metastases. Thus, we believe that the high sensitivity of [^18^F]DCFPyL, as indicated in the current study, appears plausible. Nevertheless, the findings of the current study are to be considered preliminary and the clinical performance of [^18^F]DCFPyL will have to be further analyzed in the future.

Another limitation can be found in the selection of patients. We did not recruit patients for both imaging series prospectively, and the selected population cannot serve as a representative sample. Due to the selection of patients with complex clinical questions (several of them being negative in [^68^Ga]Ga-PSMA-HBED-CC PET/CT), it might be possible that the diagnostic accuracy of [^68^Ga]Ga-PSMA-HBED-CC PET/CT is underestimated.

Comparison of the two tracers has not been carried out under identical conditions, *i.e.*, mean injected dose and start of acquisition have been higher respectively later for [^18^F]DCFPyL, as compared to [^68^Ga]Ga-PSMA-HBED-CC PET/CT. It cannot be excluded that the performance of [^68^Ga]Ga-PSMA-HBED-CC PET/CT would have been more similar to [^18^F]DCFPyL under more identical examination conditions. However, it was the aim of this study to compare the two tracers under the circumstances usually available in clinical application. Generally, lower doses and earlier acquisition periods p.i. are selected for ^68^Ga-labeled tracers. The time period of 1 h between injection of [^68^Ga]Ga-PSMA-HBED-CC and the start of PET/CT was reported by many publications [2–5].

It was beyond the scope of an observational study to show time-activity curves. The time period of 2 h between the injection of [^18^F]DCFPyL and the start of data acquisition was chosen in the light of conventional receptor imaging in nuclear medicine using other tracers with contrast enhancement on delayed images and is in accord with the data including time-activity curves published by Szabo and colleagues [14]. Szabo *et al.* have performed PET imaging at five different time points and demonstrated a small number of lesions which became visible only on PET-5 131–167 min p.i. The authors recommended delayed imaging at 2 h [14]. We have not tested different time periods between the injection of [^18^F]DCFPyL and the start of data acquisition.

Independently from the radiolabeling with [^18^F] or [^68^Ga], previous cancer therapies and low PSMA expression caused by tumor heterogeneity [16, 17] might be responsible for false-negative PET/CT results in some patients. We have learned from other studies that imaging of the choline transport and phosphorylation may detect PSMA-negative metastases in some cases [2, 3]. Future studies should investigate the diagnostic accuracy of F-18- or Ga-68-labeled PSMA in the early PSA-relapse <1 ng/ml, when [^11^C] or [^18^F] choline have low detection rates [18, 19] and should redefine the therapeutic impact of PET/CT [20–22]. Improved diagnostic accuracy should be confirmed by systematic comparison of sensitivity and specificity of both agents.

## Conclusion

Our results indicate that the [^18^F]-labeled compound [^18^F]DCFPyL is a highly promising alternative to [^68^Ga]Ga-PSMA-HBED-CC for PSMA-PET/CT imaging in relapsed prostate cancer. Based on significantly higher SUV values in the PSMA-avid lesions, [^18^F]DCFPyL PET/CT may represent a valuable tool to detect small prostate cancer lesions with high sensitivity.
